# Contextual and individual level factors influencing nutritional program effectiveness in HIV care setting in Tigray region, northern Ethiopia: Mixed methods study

**DOI:** 10.1371/journal.pone.0231859

**Published:** 2020-04-27

**Authors:** Fisaha Tesfay, Anna Ziersch, Lillian Mwanri, Sara Javanparast

**Affiliations:** 1 Southgate Institute for Health, Society and Equity, Flinders University- Australia, Bedford Park, Australia; 2 School of Public Health, Mekelle University–Ethiopia, Addis Ababa, Ethiopia; 3 College of Medicine and Public Health, Flinders University-Australia, Bedford Park, Australia; Addis Ababa University School of Public Health, ETHIOPIA

## Abstract

**Introduction:**

Addressing malnutrition is one of the key components of HIV care among people living with HIV. Since 2010, a nutritional program has been implemented to address malnutrition amongst HIV patients in Ethiopia, with patients enrolled in the program for 3 months (for mild acute malnutrition) and 6 months (for severe acute malnutrition). However, utilisation and effectiveness of the nutritional programs remain unexplored. This study aimed to examine individual level determinants and contextual factors influencing the effectiveness of the nutritional program in the Tigray region of Ethiopia.

**Methods and setting:**

The study employed a mixed-methods approach involving quantitative and qualitative research methods. In the quantitative phase of the study, records from 1757 adult patients, including socio-demographic characteristics, clinical and nutritional program outcomes were retrieved from three selected hospitals in the Tigray region, Ethiopia. Logistic regression analysis was used to identify the individual demographic and socioeconomic, clinical and immunological, and anthropometric and nutritional determinants of nutritional outcomes. The qualitative study included 33 individual interviews with adult patients, health providers, and program managers. Interview data were analysed using a framework analysis approach.

**Results:**

Amongst study participants, 55.3% (95% CI = 53.2‒57.4) recovered from malnutrition, 19% (95% CI, 17.3‒20.7) did not complete the program, and 21% (95% CI = 19.7‒23.4) completed the program but failed to recover from malnutrition. In the multivariable logistic regression analysis, those who were: living in urban areas (AOR = 1.44, 95% CI = 1.05‒1.97), employed (AOR = 1.39, 95% CI = 1.01‒1.93), attending Shul (AOR = 4.6, 95% CI = 3.15‒6.71) and Lemlem Karl (AOR = 2.5, 95% CI = 1.69‒3.71) hospitals, in clinical stages II (AOR = 2.49, 95% CI = 1.59‒3.91) and III (AOR = 1.46(1.02‒2.07), on ART for less than six months (AOR = 1.61, 95% CI = 1.09‒2.39), anaemic (AOR = 1.77, 95% = 1.29‒2.41), and diagnosed with severe acute malnutrition at enrolment (AOR = 6.43, 95% CI = 4.69‒8.3); were less likely to complete the program. Results for those who completed the program indicated that urban residence, (AOR = 1.46, 95% CI = 1.4‒2.91), attending Shul (AOR = 2.92, 95% CI = 2.04‒4.19) and Lemlem Karl (AOR = 1.49, 95% CI 1.05‒2.11) hospitals, having bedridden functional status (AOR = 0.36, 95% CI = 0.15‒0.83), advanced WHO clinical stage (WHO clinical stage IV) (AOR = 0.52, 95% CI = 0.28‒0.98) and severe malnutrition at enrolment (AOR = 4.25, 95% CI = 3.02‒5.98)) predicted non-response to the nutritional program. Qualitative interviews revealed that the taste and perceived side effects of the nutritional supplement provided as part of the nutritional program, sharing/selling practices, religious and sociocultural issues, distance and poor access to the health services were barriers to program utilisation. Nutritional counselling and health service-related factors such as a previous enrolment in the program and positive experience in the health service were enablers of program utilisation.

**Conclusion:**

There was a clear nexus between contextual factors such as distance, quality of health service and sociocultural factors, and individual patient characteristics with the effectiveness of the nutritional program. Taking individual and contextual factors into consideration in program design, planning and implementation is essential if the nutritional program in HIV care services is to achieve its goal in addressing malnutrition amongst people living with HIV.

## Introduction

Malnutrition is highly prevalent among HIV patients [1, 2] and has a negative impact on HIV care and outcomes such as adherence to treatment [3–5] and mortality [6–8]. The rate of malnutrition amongst HIV patients is particularly high in some African countries including Kenya (63%) [9], Ethiopia (46%) [10] and Brazil (43%) [11].

The integration of nutritional programs into HIV care is recommended by the World Health Organisation [12] and stipulated in national and multinational policy frameworks [12, 13], including in sub-Saharan Africa [14]. Addressing malnutrition in HIV care settings takes different forms in different contexts [15, 16]. Nutritional programs mainly involve nutritional assessment, counselling, and support using a variety of supplements, ready to use or fortified foods [17, 18].

Evidence suggests that nutritional programs have a positive impact on improving HIV care, treatment outcomes, and nutritional status among people living with HIV. According to a study conducted in Ethiopia, adults living with HIV who received nutritional support were twice as likely to achieve normal nutritional status (BMI≥18.5 kg/m^2^) than those taking ART only [19]. In addition, nutritional care using ready-to-use therapeutic foods has shown to be effective in restoring weight and improving overall health status in Malawi [20]. Other studies from Zambia [21, 22] and Haiti [23] also highlighted that providing various forms of nutritional support to people living with HIV improved adherence to ART, reiterating the key benefits and importance of nutritional care and support for people living with HIV.

In 2010, the Ethiopian Government introduced a nutritional care and support program as an integral component of the HIV care and support services [24]. The 2013–2015 National Nutritional Program of Ethiopia emphasized the need for a sustained integration of nutritional assessment, counselling and support (NACS) into the HIV treatment, care and support services [25, 26].

The nutritional support component of the nutritional program in Ethiopia provides a Ready to Use Therapeutic Food (RUTF), Plumpynut® paste for severe acute malnutrition and a Ready to Use Supplementary Food (RUSF), Plumpy’Sup® paste for moderate acute malnutrition [19, 27]. The provision of nutritional support is based on objective physical measurement (body mass index (BMI<18.5 kg/m^2^) [19, 24]. In the HIV care service, patients are assessed for malnutrition during their regular visits to HIV services. Socio-demographic, clinical, immunological and anthropometric characteristics are also collected during these visits. After a first nutritional assessment, adults with a BMI of <18.5kg/m^2^ are enrolled in the nutritional program for 3 to 6 months depending on the severity of malnutrition. Prescriptions for nutritional support are renewed monthly during the ART or separate appointments.

Despite the reported benefits of nutritional programs in HIV care outcomes [28–31], concerns about program effectiveness still remain. Studies in Ethiopia and Kenya have identified a lack of patient understanding of the nutritional program and the impact of individual demographic, socioeconomic, anthropometric and nutritional characteristics on program ineffectiveness [32–34].

An early evaluation of the nutritional program in Ethiopia, Sadler et al, (2012) found a high degree of program incompleteness (70.6%), non-response (58%) and relapse of undernutrition (20%), but the factors contributing to program failure were not comprehensively examined [19]. This evaluation identified a number of patient and health services as barriers to program effectiveness including patient socioeconomic status, clinical characteristics, and poor program understanding [19].

However, none of these studies examined individuals’ knowledge and understanding of the program and broader contextual factors that may influence the utilisation and outcomes of the nutritional program [32, 33, 35]. As such, there is little evidence on the broader demographic and socioeconomic, immunological and clinical, nutritional and anthropometric correlates of program incompleteness, and non-response among those enrolled in the nutritional program in Ethiopia.

Guided by the socio-ecological model (SEM), this study sought to address this gap by examining both the individual and contextual challenges to the effectiveness and utilisation of the nutritional program in HIV care settings in the Tigray region of Ethiopia.

The SEM is a comprehensive model that examines the complex interaction of factors that influence behaviours at multiple levels of influence including individual, interindividual, institutional and social policy levels [36, 37]. The model was used to direct the study by focusing beyond solely individual factors that may influence program utilisation and outcomes, to also examine higher-level structural factors that shape these individual factors and consequently impact program outcomes.

## Methods and materials

### Description of research settings

The study was conducted in the Tigray region, Northern Ethiopia. The predicted prevalence of HIV in the region for the year 2018 was one of the highest (1.7%) with, nearly 60,000 people in the area living with HIV [38, 39]. The researcher’s past working experience and familiarity with the region was also considered as a reason for increasing the study feasibility.

There are 37 hospitals in the Tigray region, of which 20 are primary district hospitals, 15 general hospitals and 2 regional referral hospitals [40]. Primary district hospitals are the first level of referral from the primary health care centres and general hospitals are the secondary level of care for patients referred from the district hospitals in their catchment area. Regional referral hospitals are tertiary levels services which provide service for patients referred from across Tigray and the neighboring regions. This study was conducted in three purposively selected general hospitals ─ one is a major city hospital (Mekelle hospital) and two relatively smaller hospitals (Shul and Lemlem Karl). All three hospitals are general hospitals and the catchment population for these hospitals is estimated to be two millions. According to an unpublished Tigray Regional Health Bureau annual report (2015/2016), total annual outpatient visits to Mekelle hospital were 221,251 individuals per year while the annual outpatient visit in Shul and Lemlem Karl hospitals were 98,533 and 96,686 individuals per year respectively.

### Research design, subjects and study period

A mixed-methods study was employed to examine the individual and contextual factors influencing the effectiveness and utilisation of the nutritional program in the HIV care setting. In this study, triangulation was used–where the qualitative and quantitative studies were conducted in parallel, with integration occurring at the point of analysis and discussion [41]. The SEM model informed the research design, with data collection tools reflecting the multiple layers of potential influence, and data analysis revealing emergent themes across levels.

In the quantitative section of the study, a retrospective cohort study design was employed. Patient records of 1757 adults living with HIV enrolled in the nutritional program from November 2010 up to July 2016 in the three above mentioned hospitals were retrieved retrospectively. Qualitative interviews were conducted with 20 adults enrolled in the program, 11 health providers and 2 program managers (April ‒August 2016). Study participants were recruited using purposive sampling, seeking to sample across age, gender and nutritional status at enrolment to recruit participants with diverse views.[42]. Interview participants were those enrolled in the nutritional program or directly or involved with managing or implementing the program.

### Data collection and quality assurance

A data abstraction checklist was prepared to retrieve information from the logbook, patient medical records and ART (Antiretroviral therapy) pharmacy databases of adults living with HIV and enrolled in the nutritional program during the study period. Extracted information comprised baseline demographic, socioeconomic, clinical, immunological, anthropometric and nutritional characteristics of participants. Nine data collectors working in the three hospitals were trained and closely monitored to assist in data collection.

For the qualitative stage of the study, an interview guide was developed covering topics influencing the nutritional program, health service, and system utilisation and other program and support related issues (S1 File). Interview questions were guided by the socioecological model reflecting the individual, interindividual, institutional, community and policy level factors impacting the nutritional program. Adults living with HIV were recruited during their regular follow up visits to the HIV care service. Information about the study was provided to potential participants by health providers and a researcher was available on site for those who expressed interested in participating. Health providers and program managers were recruited in their workplace via an invitation letter on a notice board. A one-on-one in-depth interview was conducted with study participants in a venue that was convenient for the participants. All interviews were audio-recorded, and comprehensive field and observation notes were taken. Participant' recruitment continued until data saturation was achieved.

### Operational definitions

The program defined nutritional program outcomes as follows:

#### Recovered

Reaching a BMI of ≥18.5kg/m^2^ for two consecutive visits within three months for moderate acute malnutrition and six months for severe acute malnutrition at baseline.

#### Program incompleteness

Dropping from the nutritional program before reaching BMI >18.5kg/m^2^.

#### Program non-response

Not reaching a BMI of ≥18.5kg/m^2^ at the end of the program.

### Data management and analysis

Quantitative data was entered into Epi-info and exported to SPSS for data management and analysis. Data were cleaned and evaluated for missing values. Missing data analysis was conducted by cross-tabulation for each variable for all the cases with negligible missing values using the Little’s completely missing at random test where the means of recorded values of each variable were compared [43]. Accordingly, the missing values were random or proportionally distributed across all categories of the recorded variables (*p = 0*.*63*). Descriptive statistics such as percentages and mean were used to describe the demographic and socioeconomic, clinical and immunological, and anthropometric and nutritional characteristics. In addition, nutritional outcomes such as recovery, program incompleteness, and non-response were described.

To identify the determinants of program incompleteness and non-response in the nutritional program, bivariate logistic regression analysis was used to estimate the crude relationship between independent and outcome variables. Variables that were statistically significant in the bivariate analysis at p<0.3 were taken to multivariable logistic regression to estimate the adjusted odds ratio (AOR) with a 95% confidence interval (CI). In the final model of multivariable logistic regression analysis, statistical significance was declared at p<0.05. Multicollinearity was checked and only number of sachets per day and baseline nutritional status were correlated. Hence, the number of sachets per day was removed from the analysis because it was not statistically significant while the baseline nutritional status was significant when the former variable was removed from the analysis.

Interviews were conducted, and audio recorded by the first author (FT) in the local language Tigrigna. Audio recordings were translated and transcribed into English by the first author (FT) which assisted with familiarisation of the data. Qualitative data were analysed using the framework approach using NVivo qualitative data analysis software [44]. After familiarisation, four purposively selected transcripts were used to generate the initial codes. A coding framework was generated inductively from the data, iteratively refined and discussed in project meetings until a consensus was reached. Codes were subsequently merged and collapsed into bigger themes and illustrative quotes were used to explain and describe the themes. Three transcripts were double-coded, and differences were discussed and agreed.

Rigour and trustworthiness in this study was maximised through measures to promote credibility and transferability. Credibility was ensured through participant and study setting triangulation. Three groups of study participants (adults, health providers, and program managers) from three different hospitals were included in the study to improve the credibility of the information. A long period of data collection (five months) created an opportunity to get participants with a diverse level of experience in the nutritional program. The use of purposive sampling, description of the study hospitals in detail and use of a similar nutritional program in other areas of Ethiopia may contribute to the transferability of the findings.

### Ethical considerations

The voluntary nature of the study was emphasised on the information sheet and participants were assured that the decision to participate or not in the study would not in any way affect service access. Interviews were conducted in a private venue to ensure confidentiality. In addition, personal identifiers of study participants were recorded, and participants were reassured about the confidential nature of the information collected. Culturally and socially acceptable and appropriate words were also used during the interview. In addition, to maintain the anonymity of study participants, pseudonyms were used in this study. Written consent was gained from all study participants.

Ethical clearance was secured from the Social and Behavioural Research Ethics Committee of Flinders University and the Ethics Committee of Mekelle University. Support letters referring to each of the study hospitals were obtained from the Tigray Regional Health Bureau (TRHB) and presented to each hospital’s administration.

## Results

### Results of the quantitative study

#### Demographic and socioeconomic characteristics of participants

The mean age of the study participants was 35.8±9.6. The majority of participants were females (63.1%) and urban residents (66.6%). With regard to education, 30.4% of participants had not attended school, 36.3% completed primary education only and 26.1% completed secondary education. Nearly half (49.6%) of the study participants were employed and working. The majority (87.8%) of participants had a family size of five and below, 81% were not members of the HIV community support group and 77.7% had disclosed their positive HIV status at least to someone (**Table 1**).

**Table 1:** Demographic and socioeconomic characteristics of study participants.

Variables	Categories	Number	Percent
Sex	Male	649	36.9
	Female	1108	63.1
Residence	Urban	1171	66.6
	Rural	586	33.4
Marital status	Never married	265	15.1
	Married (In. defacto)	722	41.1
	Separated	164	9.3
	Divorced	381	21.7
	Widow/Widower	225	12.8
Education status	No education	535	30.4
	Primary	637	36.3
	Secondary	459	26.1
	Tertiary	126	7.2
Employment	Working	871	49.6
	Employed but not working due to ill health	111	6.5
	Unemployed	732	42.7
Household family size	≤5	1543	87.8
	>5	207	11.8
Have children	Yes	1346	76.6
	No	411	23.4
Membership of HIV related community support group	Yes	333	19.0
	No	1424	81.0
Disclosure to at least someone	Yes	1366	77.7
	No	391	22.3

#### Clinical, immunological, nutritional and anthropometric characteristics

The majority (83.8%) of study participants were working or apparently healthy, 50.5% were in WHO clinical stage I or did not have the worst clinical conditions. Almost all (94.3%) participants were on ART, of which 64% had been on ART for more than 24 months. A quarter of participants had opportunistic infections with Tuberculosis the most common infection (**Table 2**).

**Table 2:** Clinical, immunological, anthropometric and nutritional characteristics of adults enrolled in the nutritional program.

Variables (n = 1757)		Number	Percent
Functional status	Working	1473	83.8
	Ambulatory	207	11.8
	Bedridden*	77	4.4
WHO clinical stage	Stage I	888	50.5
	Stage II	225	12.8
	Stage III	518	29.5
	Stage IV	126	7.2
Baseline CD4 count	<200	675	39.0
	200–349	488	28.2
	350–500	294	17.0
	>500	274	15.8
Presence of anaemia	Anaemic	687	45.7
	Not Anaemic	815	54.3
ART status	Pre-ART	100	5.7
	ART	1657	94.3
Duration on ART	<6 months	324	19.6
	6 months to 12 months	99	6.0
	12–24 months	175	10.6
	>24 months	1059	63.9
Cotrimoxazole prophylaxis	Yes	1297	73.8
	No	460	26.2
Presence of opportunistic infection	Yes	436	24.8
	No	1321	75.2
Type of opportunistic infection	TB	152	37.3
	TB and others	47	11.5
	Others	208	51.1
Nutritional status at enrolment	Mild acute undernutrition	253	14.4
	Moderate acute under nutrition	1098	62.5
	Severe acute undernutrition	406	23.1
Number of sachets/ day	≤ 3	1362	77.6
	≥4	394	22.4
Nutritional outcome	Graduated/recovered	971	55.3
	Non-respondent	379	21.0
	Incomplete (Defaulted)	329	18.7
	Death	35	2.0
	Transferred out	43	2.4

* Bedridden functional is a worest clinical idicators or severe illness among people living with HIV.

At enrolment to the nutritional program, 62.5% of participants had acute moderate malnutrition (BMI) <18.5 and >16 kg/m^2^) while 23.1% had severe acute malnutrition (BMI ≤16kg/m^2^). The majority of participants (77.6%) were prescribed three sachets per day of Plumpy’Sup® paste or Plumpynut® paste respectively for moderate and severe acute malnutrition. Regarding the nutritional outcomes, nearly half of the participants (55.3%) had ‘recovered’ (BMI>18.5kg/m^2^), 18.7% had not completed the program, and 21.0% had completed the program but had not recovered (i.e. were non-respondents). Two percent had died (2%) and a further 2.4% had transferred out of the program (**Table 2**).

#### Nutritional program incompleteness

Demographic and socioeconomic characteristics associated with nutritional program incompleteness in bivariate analysis were: gender, residence, educational status, marital status, employment, disclosure of HIV status and place of service attendance (hospital). Additionally, the clinical and immunological characteristics related to nutritional program incompleteness were: baseline WHO clinical stage, functional status, ART status, duration on ART, baseline CD4 count, anaemia and nutritional status (**Table 3**).

**Table 3:** Bivariate and multivariable analysis of factors that contribute to program incompleteness.

Variable		Nutritional program completeness		Crude Odds ratio (95% CI	Adjusted Odds ratio (95% CI)	P-value
		Completed	Not completed			
		No (%)	No (%)			
Gender	Male	485(78.9)	130(21.1)	1.12(0.91‒1.49)	1.23(0.87‒1.70)	0.22
	Female	865(81.3)	199(18.7)	1	1	
Residence	Urban	929(82.5)	197(17.5)	1.48(1.15‒1.90)	1.44(1.05‒1.97) *	0.023
	Rural	421(76.1)	132(23.9)	1	1	
Marital status	Single	210(83.0)	43(17.0)	1	1	
	Married	554(80.2)	137(19.8)	1.21(0.83‒1.76)	1.30(0.80‒2.12)	0.29
	Divorced	414(79.8)	105(20.2)	1.24(0.84‒1.83)	1.16(0.69‒1.94)	0.58
	Widowed	172(79.6)	44(20.4)	1.25(0.78‒2.0)	1.66(0.90‒3.05)	0.1
Educational status	No education	383(76.8)	116(23.2)	1.56(1.15‒2.12)	1.35(0.72‒2.53)	0.35
	Primary education	490(80.3)	120(19.7)	1.26(0.93‒1.69)	1.04(0.56‒1.92)	0.91
	Secondary education and above	477(83.7)	93(16.3)	1	1	
Employment status	Working	643(77.4)	188(22.6)	1.52(1.18‒1.97)	1.39(1.01‒1.93)	0.046
	Employed but not working due to ill health	81(81.8)	18(18.2)	1.16(0.67‒2.00)	0.80(0.39‒1.65)	0.54
	not employed and working	594(83.9)	114(16.1)	1	1	
Disclosure of HIV status	Yes	1037(79.5)	267(20.5)	1.3(0.96‒1.76)	1.23(0.83‒1.82)	0.295
	No	313(83.5)	62(16.5)	1	1	
Place of service attendance	Mekelle	907(87.7)	127(12.3)	1	1	
	Shul	215(62.7)	128(37.3)	4.25(3.19‒5.67)	4.6(3.15‒6.71) *	0.0001
	Lemlem Karl	228(75.5)	74(24.5)	2.32(1.68–3.20)	2.5(1.69‒3.71) *	0.0001
Functional status	Working	1167(82.3)	251(17.7)	1		
	Ambulatory	135(69.2)	60(30.8)	2.07(1.48‒2.88)	1.25(0.78‒2.0)	0.37
	Bedridden	48(72.7)	18(27.3)	1.74(1.00‒3.05)	0.71(0.31‒1.67)	0.44
WHO clinical stage	WHO stage I	743(85.3)	128(14.7)	1	1	
	WHO Stage II	153(71.8)	60(28.2)	2.28(1.6‒3.24)	2.49(1.59‒3.91) *	<0.001
	WHO stage III	366(76.4)	113(23.6)	1.79(1.35‒2.378)	1.46(1.02‒2.07) *	0.037
	WHO stage IV	88(75.9)	28(24.1	1.85(1.16‒2.94)	1.11(0.6‒2.01)	0.75
CD4 count	<200	469(74.4)	161(25.6)	2.82(1.84‒4.30)	1.25(0.73‒2.16)	0.41
	200–350	394(83.5)	78(16.5)	1.63(1.03‒2.56)	1.05(0.61‒1.81)	0.87
	349–500	235(82.7)	49(17.3)	1.71(1.05‒2.80)	1.27(0.71‒2.3)	0.42
	>500	238(89.1)	29(10.9)	1	1	
Haemoglobin	Anaemic	493(76.0)	156(24.0)	2.05(1.56‒2.70)	1.77(1.29‒2.41) *	<0.001
	Non-anaemic	686(86.6)	106(13.4)	1	1	
ART status at enrolment	Not on ART	66(70.2)	28(29.8)	1.81(1.14‒2.87)	1.42(0.73‒1.84)	0.71
	on ART	1284(81.0)	301(19.0)	1		
Duration on ART at enrolment	< = 6 months	210(70.0)	90(30.0)	2.42(1.79‒3.27)	1.61(1.09‒2.39)	0.018
	7–12 months	73(78.5)	20(21.5)	1.55(0.92‒2.61)	1.30(0.69‒2.46)	0.41
	13–24 months	126(77.8)	36(22.2)	1.61(1.073‒2.43)	1.30(0.79‒8.68)	0.31
	>24 months	875(85.0)	155(15.0)	1	1	
Cotrimoxazole prophylaxis	Yes	971(78.5)	266(21.5)	1.65(1.22‒2.22)	0.93(0.6‒1.10)	0.76
	No	379(85.7)	63(14.3)	1	1	
Baseline nutritional status	Mild and moderate acute malnutrition	1159(87.9)	159(12.1)	1	1	
	Severe malnutrition	191(52.9)	170(47.1)	6.49(4.98‒8.46)	6.43(4.69‒8.3) *	p<0.001

*P-value≤0.05.

In multivariate analysis, sociodemographic characteristics that had a statistically significant relationship with nutritional program incompleteness were: residence, employment, and place of service attendance (**Table 3**). When adjusting for other variables: those living in urban areas were less likely to complete the nutritional program than those from rural areas. In addition, those who were working were less likely to complete the nutritional program than those who were not working at enrolment. Regarding the relationship between hospital of attendance and program incompleteness, those who were enrolled in Shul and Lemlem Karl hospitals were less likely to complete the nutritional program than those who were enrolled in Mekelle hospital.

The clinical characteristics that had a statistically significant relationship with nutritional program incompleteness were: WHO clinical stage, anaemia at baseline and baseline nutritional status (**Table 3**). Individuals in WHO clinical stage II and III were more likely to fail to complete the nutritional program than those who were in WHO clinical stage I. In addition, those individuals who were anaemic at enrolment were less likely to complete the nutritional program than those who were not. Those who had been on ART for less than six months were less likely to complete the nutritional program than those who had for more than 24 months. Baseline nutritional status predicted program incompleteness among adults living with HIV. Thus, those with severe acute malnutrition were 6.4 times more likely to not complete the nutritional program than those enrolled with mild and moderate acute malnutrition.

#### Program non-response

Demographic and socioeconomic characteristics that were statistically significant in the bivariate logistic regression analysis were place of residence, marital status, educational status, employment, children, disclosure of HIV status and place of service attendance. In the bivariate logistic regression, the clinical and immunological determinants of non-response to the nutritional program were: functional status, WHO clinical stage, duration on ART, type of opportunistic infections and baseline nutritional status (**Table 4**).

**Table 4:** Bivariate and multivariate analysis of factors that contribute to program non-response.

Variables		Nutritional status		Crude OR (95% CI)	Adjusted OR (95%CI)	P-value
		Recovered	Non-respondent			
		No	No (%)			
Sex	Male	349(72.0)	136(28.0)	0.98(0.78‒1.28)	1.01(0.75‒1.36)	0.94
	Female	622(71.9)	243(28.1)		1.0	
Residence	Urban	315 (74.8)	106(25.2)	1.24(0.94‒1.61)	1.46(1.4‒2.91) *	0.033
	Rural	656 (70.6)	273 (29.4)	1.0	1.0	
Marital status	Never married	153(72.9)	57(27.1)	0.96(0.61‒1.51)	1.91(0.53‒1.57)	0.94
	Married	412(74.4)	142(25.6)	0.89(0.61‒1.31)	0.87(0.57‒1.31)	0.42
	Divorced	282(68.1)	132(31.9)	1.21(0.82‒1.79)	1.15(0.75‒1.77)	0.68
	Widowed/widower	124(72.1)	48(27.9)	1.0	1.0	
Educational status	No education	272(71.0)	111(29.0)	1.0	1.11(0.79‒1.58)	0.54
	Primary	348(71.0)	142(29.0)	1.0 (0.75‒1.34)	1.06(0.77‒1.45)	0.54
	Secondary and above	351(73.6)	89(28.1)	0.76(0.55‒1.04)	1.0	0.73
Employment	Unemployed	446(75.1)	148(24.9)	0.76(0.60‒0.97)	0.96(0.71‒1.21)	0.73
	Employed	54(66.7)	27(33.3)		1.0	
Husban and wife have children	No	230(71.4)	92(28.6)	1.00	1.0	
	Yes	741(72.1)	287(27.9)	1.32(0.99‒1.77)	0.87(0.61‒1.23)	0.36
Disclosure of HIV status	No	229(73.2)	84(26.8)	1.0	1.0	
	Yes	742(71.6)	295(28.4)	1.08(0.82‒1.44)	1.21(0.87‒1.67)	0.26
Place of service	Mekelle	695(76.6%)	212(23.4)	1.0	1.0	
	Shul	119(55.3)	96(44.7)	2.65(1.94‒3.61)	2.92(2.04‒4.19) *	P<0.0001
	Lemlemkarl	157(68.9)	71(31.1)	1.48(1.08‒2.04)	1.49(1.05‒2.11) *	P<0.0001
Functional status	Working	849(72.8)	318(21.2)	1.00		
	Ambulatory	88(65.2)	47(34.8)	1.43(0.98‒2.08)	1.09(0.71‒1.670)	0.57
	Bed ridden	34(70.8)	14(29.2)	1.10(0.58‒2.08)	0.36(0.15‒0.83) *	0.02
WHO clinical stage	Stage I	552(74.3)	191(25.7)	1.0	1.0	
	Stage II	108(70.6)	45(29.4)	1.20(0.82‒1.77)	1.02(0.65‒1.60)	0.56
	Stage III	245(66.9)	121(33.1)	1.43(1.09‒1.88)	1.03(0.74‒1.44)	0.37
	Stage IV	66(75.0)	22(25.0)	0.96(0.58‒1.60)	0.52(0.28‒0.98)”	0.05
Duration on ART	>24 months	624(71.3)	251(28.7)	1.0	1.0	
	13–24 months	92(73.0)	34(27.0)	0.92(0.60‒1.40)	0.85(0.54‒1.37)	0.39
	6–12 months	57(78.1)	16(21.9)	0.7(0.39‒1.24)	0.66(0.35‒1.24)	0.18
	<6 months	152(72.4)	58(27.6)	0.95(0.68‒1.33)	0.85(0.58‒1.26)	0.47
Nutritional status at baseline	Mild and moderate malnutrition	885(76.4)	274(23.6)	1.0	1.0	
	Severe malnutrition	86(45.0)	105(55.0)	3.94(2.88‒5.41)	4.25(3.02‒5.98) *	P<0.0001

In a multivariate logistic regression analysis, residence and place of service attendance were the demographic and socioeconomic characteristics that were related to non-response (**Table 4)**. Those who were from an urban area at the time of enrolment to the nutritional program were 1.5 times more likely to be non-respondent than those from rural areas. In addition, those who attended the nutritional program in Shul and Lemlem Karl hospitals were 3 and 1.5 times more likely to fail to respond to the program than those who attended the program in Mekelle hospital.

In the multivariate analysis baseline functional status, WHO clinical stage and nutritional status were the clinical and nutritional characteristics that independently predicted non-response to the nutritional program. In an adjusted analysis, individuals who were bedridden and in WHO clinical stage IV were more likely to respond to the program than those who were working (apparently healthy) and in WHO clinical stage I respectively. Those, with severe acute undernutrition at enrolment, were 4 times more likely to be non-respondent than those who were moderate or mild acute malnourished.

### Results of the qualitative study

#### Characteristics of interview participants

A total of 20 adults and 13 program staff (11 health providers and 2 program managers) were interviewed. Adult participants included eight males and twelve females. Twelve adult participants resided in urban and eight in rural areas. With regards to the educational status of adult study participants, ten attended primary school, six with secondary and above, and four reporting no education. Marital characteristics of study participants included six who reported being married, six who were single, five were widowed and three were divorced. Of the program staff interviewed, seven of the program staff interviewed were females and the remaining six were males. All program staff had Bachelor degree or above and 11 were married.

#### Barriers to and facilitators of nutritional program utilisation

Identified barriers to successful utilisation of the nutritional program included the taste and side effects of the nutritional support, sharing and selling practices, the way that stigma and discrimination were potentially enhanced by the program, religious and sociocultural issues, distance and access to services, and poverty and food insecurity. Emergent program utilisation facilitators were some elements of the nutritional counselling, positive experiences of health services and also previous experience in the program. These themes are discussed further below.

#### Barriers to the utilisation of the nutritional program

Two types of nutritional supports were used in the nutritional program‒Plumpynut for severely acute malnourished and Plumpysup for moderately acute malnourished adult HIV patients. Participants, in general, reported Plumpynut as having a tolerable taste. However, the sugary and salty taste and oily character was a source of complaints among some participants and contributed to defaulting from the nutritional program. Some reported differences in the taste between the two ‘brands’ of Plumpynut with one being less tolerable. Furthermore, the Plumpysup was saltier than the Plumpynut.

The Plumpynut that I took at the beginning was so nice but the one I took recently was too salty and it is very difficult to consume it. Because it is too salty, it is not easy to consume and sometimes I couldn’t eat two sachets per day. The first ration of the Plumpynut, I took it properly and it was very good, but the subsequent rations were too salty and difficult to consume (Adult female, age 25 #17).

Side effects related to the nutritional supports were also reported by study participants. For instance, general discomfort, gastric upset, and gastritis, nausea, vomiting, and diarrhea following the consumption of both types of nutritional support were reported by participants.

The other problems associated with this [the nutritional support] are also related to complaints such as gastric upset, sugary and salty taste (Health provider, age 29 #3).

Sharing of nutritional support was a common practice among participants. A range of factors such as cultural issues, poor livelihood, and food insecurity were the drivers of this sharing. Sharing of the nutritional support was particularly pronounced among mothers and was also seen as a good mechanism to hide positive HIV status among family members:

*Yes, there is sharing among household members. As far as there is an economic problem, it is not necessarily selling but also sharing. Because if it is given to the child [referring to a caregiver and her child], it is likely that the mother will share it with his siblings. If given two sachets then the mother gives one to her other child and keeps one for the HIV positive child (Health provider, age 35 #7)*.

*Instead of eating it for himself only [referring to a caregiver and her child], they want to sell and change it for other household needs such as sugar, salt or oil. Most of the time the reason is this but the base is the poor livelihood condition. So, they [the household or family of the child] are not doing it [selling] intentionally but it (selling) is because of their economic problems (Health provider, age 39 #9)*.

The issues around selling the nutritional support was a significant concern as noted by health providers because of potential delays to recovery and its contribution to program incompleteness. Program participants were aware of these potential negative impacts:

It [selling of the nutritional support] would have negative consequences to me. Because I was given to replace my weight loss and if I sell it [the nutritional support] I may not improve my weight. You may also get sicker and sicker which will result in further illness (Adult female, age 20 #10).

However, there were no study participants reporting selling of the nutritional support themselves, though they did say they knew of others who did and also reported its availability in local shops, despite it is being a medical product that should only be available in licensed drug stores. A range of reasons for selling such as addiction, poverty, boredom with the nutritional support, and a lack of knowledge and understanding of the use of the support were also reported.

#### Stigma and discrimination associated with HIV disclosure and nutritional program utilization

Disclosure of positive HIV status and associated stigma are often a challenge for people living with HIV. A number of study participants reported disclosure of their positive HIV status only to family members and some were reluctant to disclose their positive HIV status to anyone including their family members, especially y those living in rural areas. Due to the limited level of HIV status disclosure among study participants, many participants believed that the nutritional program enhanced HIV related stigma. They reported that as the nutritional support is closely linked to HIV by the community, the use of nutritional program increases the risk of disclosure and associated stigma:

*I live like what I was before means HIV negative. Even when I take the Plumpynut, I don’t want to show it to anyone at all. I consume it [the plumpynut] and bring it [the plumpynut] back to here (the health facility) because I don’t want to be seen. (Adult female, age 29 #15)*.

*Yes, they [community] relate it. I am sure they relate it. But since it says food by prescription, it is obvious that it is for malnourished individuals. Since it [the plumpynut] is taken from the HIV clinic the community knows it [HIV condition] by that. (Adult male age 47 #22)*.

*When using, I close my door to use my Plumpynut and ART medication. Why I did this is to avoid stigmatization. Because if they (neighbours) know, they will not allow me to rent their house or use toilets. So, if this happens I have nowhere to go. So, the only option I have to do is to use it like this [hiding] (Adult female, age 29 #15)*.

Other factors that participants reported to increase the risk of disclosure and associated stigma were an increase in the frequency of health service visits for those who were enrolled in the program and carrying or consuming nutritional support with its distinct packaging from the health facility to their home.

If some know that you [referring to himself] are using the medication and Plumpynut because of your HIV status, there may be problems. So, people usually hide the Plumpynut during transportation and at their home especially when they live in a house rent (Adult male, age 54 #7).

A small minority were more open about their status and were not concerned about the nutritional program and potential contributions to stigma and discrimination:

All my family members know about my HIV status including my children, brothers, and sisters. I also take my medication and the Plumpynut without fear, anytime and anywhere. Yes, my friend knows. (Adult male, age 52 #5).

#### Religious and sociocultural issues

The nutritional support contains contents that are religiously prohibited to consume during fasting and some participants were, therefore, reluctant to consume it during the fasting periods. These individuals believed that consuming the nutritional support during fasting time is a sin, which might worsen their HIV condition and perhaps facilitate death. This was particularly reported by older participants, due to the widely held community belief that older people should fast more than anybody else.

*When they [the community] saw you while eating and consuming non-fasting foods, it may be unacceptable for the community. Because in our society old people should fast (Adult male, 54 #7)*.

*About fasting, I usually didn’t take the Plumpynut on Wednesday and Friday. I also didn’t eat during the fasting for Easter. So, except Wednesday and Friday, I usually continue to eat the Plumpynut in the other days because of the ART medication (Adult male, age 29 #8)*.

Health providers likewise highlighted fasting as an important reason for the interruption of the nutritional program.

*Old women especially those highly related to religion, they don’t prefer to take it, especially during fasting. They [old women] refuse to take generally packaged foods. Even if we (referring to people enrolled in the program] try convincing them, they usually relate it with religion and didn’t take the food (Health provider, age 30 #10)*.

*If the time they are diagnosed malnourished is during the time of fasting, they may not take the Plumpynut at all. This works similarly in Muslim and Christians, if the food is given during their fasting time, they didn’t take it at all. So, because of this, they may not take it properly (Health provider, age 39 #9)*.

On the other hand, there were some participants who reported consuming the nutritional support (Plumpynut) at any time even during the fasting period after discussing the issue with their religious leaders.

#### Distance and access to services

Enrolment in the nutritional program requires more frequent visits to the health facility for monitoring nutritional status and for collection of the nutritional support. Nutritional support is only provided in specific treatment centres and so patients need to travel to these centres. Participants reported that traveling to collect nutritional support every month was difficult because they could not afford the transportation cost and had difficulties traveling due to ill health.

That is my only problem (transportation fee) because I didn’t have anybody to support me. I have to feed my kids and no one is helping me. So, transportation is the most important concern when I come here (Adult female, age 37 #16).

Furthermore, health providers reported that some program participants moved temporarily to other areas (for employment) after enrolment in the nutritional program and that there were no similar services in these areas for participants to visit.

There are many defaulters because some went to traditional gold mining and farming to western Tigray. But the nutritional program doesn’t consider this when a patients moves to other places employment and job related activities (Health provider, age 40 #1).

#### Poverty, food insecurity and the sharing of nutrition support for family meals

Many participants enrolled in the nutritional program reported problem accessing adequate food in terms of quantity as well as quality or ‘food balance, due to a lack of adequate sources of income:

Made an effort to maintain my weight but your [referring to herself] economic status determines your nutritional status. I didn’t have enough, then from where can I get? I try by what I have but my economic status is very low. I know if you eat properly, I will have the energy to work and perform like others but if you are poor, you can do nothing. I live with whatever I have (Adult female, age 48 #9).

The economic circumstances of adults in this study were fragile. For instance, a number of adults had either no education or their school attendance was low, with no or limited skills. Limited skills contributed to minimal job and life opportunities and led to a lack of reliable income. Thus, lack of regular and reliable employment, as well as the absence of sustainable and reliable income to support them and their family, contributed to poverty and poor livelihood.

If you (respondent referring to herself) are poor and don’t have a good job, even if you try to create your own job, no one allows you to work because of no education. I asked the Kebele administration for a job, but they told me that you have no education. This makes I very angry. Otherwise, if I have a job, I will eat adequate and balance food. (Adult female, age 31 #1).

Due to food insecurity within households, adults reported sharing the nutritional support as family meals, which meant that they would not have adequate consumption of their ration

I am not taking adequate food and even now, I can’t get enough. I give everything I have to my children and I focus on feeding and care for them [referring to her children]. With the problems I have, I can’t get enough food to support myself and my children. (Adult female, age 29 #15).

Food insecurity was also a reason for non-response and default from the nutritional program. Some adults were reluctant to graduate because the nutritional support was used as a source of income to fulfill household necessities.

Even though, the patients were counselled well, one reason for selling could be the existing economic problems. Poverty by itself would encourage individuals to sell it [the nutritional support] and spend the money on something that matters to the family is there (Program manager, age #1).

Enablers of utilisation of the nutritional program. *Nutritional counselling*. Nutritional counselling was found to be important in improving patients’ knowledge about the program and broader eating behaviour. The counselling program was meant to cover the use of nutritional support, dietary diversification, hygiene, and sanitation. While most reported the counselling received was largely related to the nutritional support including how to use and its benefits, there was a common perspective that counselling sessions assisted them in utilising the nutritional support, including the importance of not selling or sharing it:

It [the nutritional counselling] is helpful. That is why I am improving my weight and overall condition. They told me that it is important to get good energy. Because the health providers tell us that it [the nutritional support] is good, you can see me thanks to god I am improving. It (the nutritional support) is good, if you eat it properly, you [participant referring to himself] will be changed and improve very well. They [referring to health providers] say “It is important and will give you strength—you have to eat it and if you can’t eat it you consume it using bread”. (Adult female, age 29 #3).

Nevertheless, health providers highlighted that not everyone had necessarily received or been able to apply all the other elements of the nutritional counselling in managing their HIV related undernutrition due to limitations associated with the nutritional counselling. For example, an individual received only one counselling session when enrolled to the program, counselling sessions were on short duration, there was a lack of counselling guideline, and health providers were not provided ongoing capacity building opportunities.

Had it been that they [referring to study participants] apply all the counselling services given to them like the ART medication, they [referring to study participants] may get the necessary benefits from the nutritional program as well we may not have default or lost to follow up from the nutritional program (Health provider, age 40 #1).

#### Positive experience of the health services

Many participants living with HIV reported trust and good relationships with health providers who were offering the service as an enabler to program utilisation. Supports provided by health providers included social and psychological support.

*Very good in all aspects and the providers are always helpful. …the health service in this clinic is very good at supporting us and providing good service (Adult female, age 29 #3)*.

*The way they [health providers] handle you is very good and excellent. Especially there is one health provider whom she is always good to me. I also didn’t hide anything from her. I tell her everything and the counselling she gave me is very important (Adult male, age 40 #18)*.

#### Previous experience of engaging in the program

Nine out of 20 adults had a history of a previous enrolment into the nutritional program but only a few of them had recovered in the past. Some participants felt previous enrolment in the nutritional program assisted them to better understand the program and consequently their current practice such as fasting during program enrollment. Furthermore, their tolerance of the nutritional support improved from their previous enrollment which also encouraged current utilisation.

During my first enrolment, I totally didn’t know about it [the nutritional program]. While I was supposed to take 2 per day I was taking three or four per day. But in the second and third enrolment, I take it (the nutritional support) based on the recommendations of the health provider (Adult male, age 47 #22).

Recovery in prior enrolments in the nutritional program had also a positive impact on those who were subsequently enrolled in the nutritional program. This also created an opportunity for positive peer influence among those enrolled in the nutritional program.

Previously, I did not eat it during fasting time. I kept all the Plumpynut until the breaking of the fasting time. I had also stopped once but now (current enrolment) I am using it properly (Adult female, age 31 #1).

Previous recovery in the nutritional program was a positive experience for adults in terms of its benefits of proper use of the nutritional program. It was an experience to which many brought positive prospects about their current enrolment in the nutritional program.

## Discussion

Findings of the current study demonstrated that the nutritional program in the Tigray region of Ethiopia is characterised by high program incompleteness and non-response. Various individual and contextual factors at different levels contributed to the ineffectiveness of the program. These nutritional outcomes and factors affecting them are discussed in relation to the socioecological model (SEM) which has helped to explain and interpret the relationship between the individual and contextual factors constraining the effectiveness of the program.

The study found that 55% of adults in the nutritional program had achieved nutritional recovery. This finding is comparable with previous studies in sub-Saharan Africa [45, 46], but contrary to a similar study in Ethiopia which found only a 33% recovery rate among adults living with HIV [19].

This study also found 19% program incompleteness and 21% non-response among adults enrolled in the nutritional program. Comparable magnitude of program failure (default plus non-response) were reported from other studies in Ethiopia (38%) [35]) and Kenya and Uganda (48%) [45]. However, these studies were slightly dissimilar to the current study as they did not report on program incompleteness and non-response separately. Another study conducted in Ethiopia reported a very high program incompleteness (71%) and non-response (58.4%) [19]. The wide difference in program effectiveness may be partly explained by the differences in context in which the program was implemented and differences in the implementation stage of the program. Additionally, the study by Sadler and colleagues (2012) in Ethiopia was conducted at the early stages of the nutritional program where health providers and program users had a minimal understanding of the program [19].

One of the key findings from this study was an understanding of the importance of how a combination of individual and contextual factors contributed to the challenges in relation to the nutritional program effectiveness in the Ethiopian context. Participants who resided in urban areas were less likely to complete the program or recover. While it might have been expected that urban setting would provide an easier access to health services including the nutritional program, factors such as high levels of poverty and the lack of income-generating activities in urban areas [47], as well as a lack of land to grow food, may contribute to food insecurity and partially explain this finding.

The hospital setting also has influenced the effectiveness of the nutritional program. Adults who attended the nutritional program at Shul and Lemlem Karl hospitals), which are in relatively distant settings from Mekelle (the capital city of Tigray region), were more likely to become non-respondent and fail to complete the nutritional program than those attending in Mekelle hospital. While this contradicts the findings above in relation to urban and rural differences these differences may relate to system-level resource allocation and distribution where health facilities located far from the place where the health service is coordinated and organised may have limitations in the distribution of the nutritional support, capacity building of health providers and strengthening of the program. Levesque et al have stated the importance of geographic location in influencing access to, and utilisation of health services where more distant health facilities may have limited access to vital resources and supplies [48]. Similar findings regarding differences in program ineffectiveness (non-response, incompleteness, and mortality) between program sites were also reported in a study conducted in Kenya and Uganda [45] but the authors did not comment on the distance of the sites.

Only a small number of clinical and immunological characteristics were associated with the nutritional outcomes. This included WHO clinical stage, baseline functional status and being on ART for more than 24 months. Advanced WHO clinical stage (II and III) was associated with program incompleteness while those with advanced WHO clinical stage (IV) were less likely to become non-respondent to the nutritional program. Moreover, findings of this study indicated that those who were bedridden ‒another clinical parameter that indicates the clinical severity of illness‒ were also less likely to become non-respondent than those who were working (apparently healthy). There are no other studies indicating a relationship between the WHO clinical stage and functional status with nutritional outcomes such as program incompleteness and non-response. However other clinical indicators such as the presence of opportunistic infections have been reported to be associated with nutritional program failure in a study in Malawi [28]. Advanced clinical HIV conditions could delay nutritional recovery and contribute to program non-response due to the clinical impacts of infection, but it has enhanced nutritional recovery in this study. It is beyond the scope of this study to explain this because of the lack of primary data and the nature of secondary data used in the current study. However, it can be speculated that those with the worst clinical conditions might have benefited from close monitoring and follow up of their clinical illness which may contribute to a better response to the nutritional program. This is an important area for further investigation.

Severe acute malnutrition at enrolment to the nutritional program was also associated with nutritional program incompleteness and non-response. This finding was supported by other studies conducted in sub-Saharan African countries [19, 49]. Severe malnutrition might imply severe clinical HIV conditions, which then would lead to poor nutritional recovery or incompleteness because of the clinical effects of the HIV condition.[50]. Severe clinical conditions can play a bidirectional impact on the effectiveness of the nutritional program. While severe clinical illness can contribute to the ineffectiveness of the nutritional program due to the clinical impact of infection, it can also create an opportunity for a better and close clinical monitoring of the nutritional program among those with severe clinical conditions.

The taste and side effects of the nutritional program were one of the barriers to program utilisation, and similar findings were reported in another study from Ethiopia and elsewhere [51, 52]. Participants in both these studies stated that the oily, salty and sugary characteristics of the nutritional support and side effects such as diarrhea and nausea were sources of key concerns.

The nutritional program in Ethiopia is designed and implemented in a context where food sharing is considered as a valuable and unique cultural identity, and a significant number of adults, particularly women, shared the nutritional support either with family members or others. Other similar studies also reported the presence of sharing practice among participants of nutritional programs [33, 51, 52]. A range of other studies have also reported more women sharing the nutritional support than men [17, 19]. Despite health providers' reports of selling the nutritional support by participants, there were no direct reports of selling experience among participants, although they were aware of the nutritional support being available in the local shops. Similar findings were found in other studies conducted in Ethiopia [33, 51] and elsewhere [52].

In areas where HIV is believed as deviance from moral values, stigma related to HIV is a common challenge [53]. Stigma and discrimination have impacts on the nutritional program at various levels [37, 54, 55] because of the community attitude towards HIV, the nutritional program and individual stigma perceptions. Qualitative evidence in this study indicated that the community commonly relates the nutritional support with HIV. Thus the nutritional support had the potential to lead to the disclosure of positive HIV status through the distinctive packaging of the nutritional support, as well as increased frequency of visits to the health facility. Other researchers from Kenya [17, 52] and Ethiopia [32], have also shown that people living with HIV using the nutritional support “Plumpynut” have faced challenges in relation to stigma and therefore were reluctant to visit health facilities and to use the nutritional support.

Ethiopia is a society where religious fasting remains the most important spiritual aspect of life [56]. Fasting in the Ethiopian Orthodox church involves abstaining from meat, egg and dairy products for a total of 190‒250 days in a year [57]. The nutritional support contains dairy products, which is prohibited from consumption during fasting. For some participants, religious fasting interfered with their consumption of the nutritional support. However, others used it as prescribed after discussions with their religious fathers. Hence, building relationships with religious fathers and leaders may assist nutritional programs to address the issue of religious fasting.

The health system context under which the nutritional program is designed and implemented also negatively impacted the utilisation of the nutritional program. Distance and transportation were fundamental challenges related to access linked to the health system. A study from Kenya also showed issues of transportation and frailty of the person receiving the nutritional support negatively impacted utilisation [52].

The nutritional program is implemented in a socioeconomic situation characterised by food insecurity, poverty, and poor livelihood. Studies elsewhere have demonstrated the susceptibility of people living with HIV to food insecurity in general [58–60], but food insecurity also acted as motivation to enrolment into the nutritional program in the current study. Additionally, studies from Ethiopia [19] and Malawi [61] reported that adults living with HIV who were food insecure were more likely to become non-respondent than their counterparts.

Evidence from a systematic review highlighted the importance of culturally sound nutritional counselling to improve the effectiveness of nutritional programs [54]. The nutritional counselling in the nutritional program was a crucial source of information about the nutritional program and use of the nutritional support. Despite challenges to nutritional counselling such as limited counselling sessions, short session duration, lack of counselling guideline, and lack of ongoing capacity building of health providers, it still seemed to facilitate utilisation of the nutritional program in HIV care.

A positive relationship with health providers facilitated program utilisation. Such relationships had enhanced trust with the health providers and preparedness by people enrolled in the program to take on the advice and recommendations they were given by them. This finding is consistent with the findings by Dawson-Rose, et al (2016) which reported that trust between health providers and people living with HIV is crucial for health service utilisation [62].

In accordance with the findings of the current study, the previous enrolment in the nutritional program enhanced the current program utilisation. Previous enrolment also seemed to improve participants’ knowledge about the existence of nutritional programs and benefits of the nutritional support. Furthermore, improved nutritional status or nutritional recovery in their previous enrolment had improved optimism of nutritional recovery during their current enrolment. Repeated experience of nutritional counselling and education related to multiple enrolments had also improved knowledge and understanding and facilitated utilisation of the nutritional program. However, multiple enrolments to the nutritional program are not at the interest of the nutritional program in HIV care settings and indicate failure of the nutritional program to prevent relapse.

This study was underpinned by the socioecological model to examine the dynamic relationship of a range of different factors that impact the effectiveness and utilisation of the nutritional program in HIV care. The key factors identified in the study often reflect overlapping elements of various factors at different levels. For example, the clinical characteristics and poor nutritional status at enrolment are individual level factors related to the individual's biological differences in responding to infection, but may also reflect institutional level factors related to the poor management of opportunistic infections at health facilities [55, 63].

Institutional level factors are related to the health institution which includes organisational structures and culture that have a critical influence on health behaviours [55, 63]. A range of institutional-level factors such as nutritional counselling and health service factors (trust) influenced utilisation of the nutritional program through a strong relationship with policy and individual level factors. Trust between the patients and health providers was an institutional level factor and can influence an individual person’s perception and attitude towards health behaviour and outcomes [64]. Nutritional counselling in the nutritional program is an institutional and policy level factor [55] and the impact of social institutions is key in influencing individual health and behaviour [63]

The taste and side effects of the nutritional support have an overarching impact on the utilisation of the nutritional program. Taste preferences are determined by the individual’s identity and food culture within the broader community level and what a certain food such as the nutritional support tastes like is shaped by the identity and food culture of the individual [65]. The taste of the nutritional support used in the nutritional program was used in HIV care for adults with no modification or contextualisation to fit into local cultures. The selection and recommendation of the nutritional support (Plumpynut/Plumpysup) go back to the policy environment in which the nutritional program is designed, an example of policy level factors that can affect people’s practices at the individual level.

Sharing and selling of nutritional support were important interindividual level factors that impacted the utilisation and effectiveness of the nutritional program. Community level factors such as stigma and discrimination, and policy levels factors such as program design and implementation had also negatively or positively impact the effectiveness of the nutritional program [55, 63, 64]. For instance, disclosure of positive HIV status and family acceptance and family support are important factors that influence the sharing and selling of nutritional support. Furthermore, despite nutritional counselling that discourages selling and sharing practice, the socioeconomic condition under which adults live is characterised by poverty and poor livelihood. In the socioecological model, formal and informal social organisations play a key role in influencing health behaviour and outcomes [63, 66]. Thus, the nutritional program was implemented within a broader socio-cultural environment where religious and cultural factors have a huge impact on community norms, eating patterns, and preferences for different foods [55, 66]. Moreover, the nutritional program was implemented in a community where enrolment in the nutritional program was closely linked with positive HIV status and enrolment enhanced to stigma and discrimination. For instance, HIV is considered as deviance from community norms and rules, and the socioecological model highlights the influence of such norms on health behaviour and outcomes [63, 66].

Policy level factors in the socioecological model include local, regional and national laws and policies including their interpretation and enforcement [63]. Access related problems are broader policy level factors because of the less decentralised service of the nutritional program to a similar extent with HIV service [67]. The nutritional program primarily has focused on treating malnutrition rather than the underlying determinants of malnutrition including poverty, poor livelihood, and food insecurity. This approach is a key policy gap that needs to be addressed in order to sustainably combat malnutrition among people living with HIV. The ineffectiveness of the nutritional program in urban areas could also be due to cross-cutting issues recognised across multiple levels of the model particularly policy and community level factors where living in urban areas may be related to selling of the nutritional support.[37, 63].

### Strengths and limitations of the study

The study was able to draw together quantitative data with qualitative material from multiple sources to investigate the multiple and overlapping factors that affect program utilisation and effectiveness. However, the quantitative study used secondary data and we could not verify the precision and accuracy of the original measurements. Thus, there may be measurement error, misclassification of outcomes such as program incompleteness and non-response. In the qualitative data, some original meaning (as narrated by the study participant) may be lost during the process of translation and transcription. However, a transcription and translation accuracy test was done and indicated a high level of accuracy. The use of an audio recording device could also have affected participants' willingness to disclose information. However, participants were reassured that the interviews were confidential and would only be used for the research purpose.

This study was unable to include primary health care centres and the effectiveness of the nutritional program in such facilities. Hence, further study should be conducted to determine the effectiveness of the nutritional program in primary health care centres to better understand issues of access and enhance further decentralisation of the nutritional program.

### Conclusion and recommendation

This study sets out to identify the individual and contextual level factors that affect nutritional programs in HIV care settings. It showed high rates of program incompleteness and non-response and identified key factors, at the individual, interindividual, institutional, community and social policy levels, that were associated with program utilisation and outcomes. The results of this study indicated that all of these levels are important considerations for nutritional programs in the HIV care setting to address the nutritional problems of people living with HIV. A key implication of the findings is that nutritional programs in HIV settings should be reoriented towards addressing the underlying challenges such as poverty, poor livelihood, and food insecurity. In addition, the involvement of religious leaders in the design and implementation of the nutritional program may also improve the effectiveness of the nutritional program. Further and detailed work needs to be done to identify how stigma and discrimination, food insecurity and nutritional counselling impact nutritional programs in HIV care settings.

## Supporting information

S1 File (DOCX)

S2 File (DOCX)

S3 File (DOCX)
