# Supplementary material for: Contextual and individual level factors influencing nutritional program effectiveness in HIV care setting in Tigray region, northern Ethiopia: Mixed methods study
Source: PLoS One. 2020 Apr 27;15(4):e0231859. doi: 10.1371/journal.pone.0231859 (PMC7185904; doi:10.1371/journal.pone.0231859)
Supplement: S1 File — (DOCX) [file pone.0231859.s001.docx]

# e For adult HIV patients

| Sociodemographic characteristics of study participants | | |
| --- | --- | --- |
| S.No | Questions | Code and category |
| Q101 | How old are you? | 31             Years |
| Q102 | What is your religion? | 1. Orthodox 2. Muslim 3. Protestant 4. Catholic 5. Others specify |
| Q103 | What is your ethnicity | 1. Tigray 2. Amhara 3. Oromo 4. Gurage 5. Others, specify |
| Q104 | Are you currently married or living together with a man/woman as if married | 1. Never married 2. Yes, currently married 3. Yes, living with a man/woman but not married 4. Widow 5. Divorced 6. Others: |
| Q105 | Who lives with you in your household and how many (including yourself)? | 4 |
| Q106 | Have you attended formal school? | 1. 1.Yes 2. 2. No |
| Q107 | If yes to Q 106  What is the highest grade you completed? | - 1. Primary   2. Secondary   3. Tertiary and above |
| Q108 | What is your current employment status? | 1. Employed 2. Not employed 3. Others specify |
| Q109 | What is your household’s average monthly income? (it means your and your spouse’s income combined) | 900               ETB |
| 110 | Average distance to reach the health facility you receive FBP service? | 1/2           Hours’ |
| 111 | How long have you been in the program? | 03       Months |

1. Can you tell me a bit about your HIV condition, family and the services you get for your HIV?

**Probing questions**

- How long has it been since your diagnosis?
- Are there other family members living with you who have HIV?
- What services/treatments are you getting for your HIV? **(Probe**: ART, nutritional assessment, nutritional counselling and provision of therapeutic/supplementary food?)

1. Okay my interest/study is around the food, your weight and nutrition problems that some HIV patients have. Can we talk a bit about how and why you got involved in the nutrition program?

**Probing questions**

- Can you recall why they recommended the program to you?
- Was your weight and nutritional status a concern to you?
- Can you tell me the reasons that you are thin/malnourished?
- How do you think your life situation like access to food and affordability attributed to poor nutritional status?
- Can we talk a bit about the reasons people or you need such therapeutic/supplementary food?

1. What has been the best services you received from this clinic in relation to the weight and nutrition problems so far? Why?

Have your nutrition condition and weight improved since you have been involved in the program? Why do you think so? If not, what are the key reasons?

1. About the food provided to you, do you use it as recommended by the health providers

**Probing questions**

- How you consume it
- Can you tell me about the taste, ration size, is it different from what you eat at home?
- When you receive the supplementary/therapeutic food, whom do you though is the other people you share with?
- Have you ever think of stopping using it (what are your reasons)?
- How you transport it to your home

1. Now, I will ask you about the nutritional counselling you get in the FBP clinic.

**Probing questions**

- How helpful is the nutritional counselling to maintain your weight?
- How does the nutritional counselling given here benefited you in relation to the supplementary/therapeutic food?
- Do you think the health provider considers your household or life conditions when providing counselling?
- Can you describe me a typical counselling session?
- Has the health providers explained to you how and when to use the supplementary/therapeutic food? Are you given the chance to ask questions or the challenges you have?

1. Ok, let’s talk about cultural , religious and others issues that might affect your use of the supplementary /therapeutic food

**Probing questions**

- Are there particular beliefs about food and malnutrition in your community? Do these beliefs influence you how you use or think about the food by prescription program?
- Does the nutritional support differ from the food you use at home?
- Who knows in your family, friends and neighbours that you are taking the supplementary or therapeutic food?
- Can knowledge by your neighbour or friends affect your continued use of the supplementary or therapeutic food? (Why?), its impact to access social services?

Do you have any problem in taking the food in front of your family, friends or neighbours, etc.?

- Can you consume the food anytime or are there times where you can’t take it?(why )

1. Does your use of the therapeutic food related to your HIV treatment? (Why and how?)
2. Have you been in the program before and what is your previous history?

- Did you recovered before and enter to the program again?

1. Have you had any difficulties being put at the program? How did you overcome them or how would you supported to overcome them?

- How do you think the program can be improved?

**Questions extracted from the transcripts**

**Counselling related issues**

- What about counselling? Is there any counselling related to this?
- With the plump nut? About how to use it, when to use it, how much you should take per day?
- What are the counselling services you obtained from this clinic about the use of plumpnut?
