# Supplementary material for: Contextual and individual level factors influencing nutritional program effectiveness in HIV care setting in Tigray region, northern Ethiopia: Mixed methods study
Source: PLoS One. 2020 Apr 27;15(4):e0231859. doi: 10.1371/journal.pone.0231859 (PMC7185904; doi:10.1371/journal.pone.0231859)
Supplement: S2 File — (DOCX) [file pone.0231859.s002.docx]

1. Can you tell me a bit about the services offered to HIV patients in this clinic?

**Probing questions**

- - - - Chronic HIV care
- ART
- Nutritional assessment
- Nutritional support
- Nutritional counselling

1. Can you describe me the specific activities of the nutritional program?
2. Ok, I am interested with the nutritional program given to HIV patients (specifically children and adults). What do you think is about the patients’ attitude and perception about the FBP?

**Probing questions**

- How and to what extent did patients use the food as prescribed?
- What is the response of the patients to the therapeutic /supplementary food? (How many improve? For those failing to improve, what are the reasons for non-response and default?
- Adults
- Children
- What mechanisms did you use to ensure that patients are using the prescribed food as recommended and how compliant are the patients to the food?
- Are there misuses of the prescribed food by patients (adult and children), what are these misuses and reasons for misuses?

1. In your view, what health service related issues negatively or positively affect the use of the nutritional program?

**Probing questions**

- What is the level of integration of the nutritional program and other HIV services (appointment date? Service provision, use of the same person, time and space for all the HIV and nutritional program services?
- Are there human power and supply related issues in the nutritional program and how does those influence the nutritional recovery?
- How did beneficiaries of the nutritional program perceive about the supplementary/therapeutic food? (Food to fulfil dietary needs, medicinal food or food to be shared among family members?)
- When do patients discontinue from the therapeutic food in the course of the nutritional therapy (**Probe**: why?)

1. How complaints are beneficiaries of the nutritional program to the nutritional counselling?

**Probing questions**

- Why do some patients fail to comply and others do?
- Can you tell me the mechanisms patients use the nutritional support?
- Have you come across any refusal of the nutritional support?(Why?)

1. How frequent and to what extent is the nutritional counselling? (Mention some general points about the counselling)?
2. Community related issues that influence use of the nutritional program (Adult and children HIV patients).

**Probing questions**

- Perceived stigma, food practice, culture, religion (fasting).
- Do you think that patients consumption of the therapeutic /supplementary food affects their interaction with community they live in (neighbours, friends) (how?)

1. As health providers, what concerns surround the HIV care and support service in general and the nutritional program in particular? (Adult and children).

**Probing questions**

- Adherence to ART and pre-ART service and its impact on the nutritional program?
- Cultural and religious appropriateness of the therapeutic /supplementary food
- About the taste, ration size, packaging and transportation

1. Now, let’s talk about the challenges of the nutritional program, what challenges and success are there about the nutritional program? In general (adult and children in particular).

**Probing questions**

- Services or program related challenges to the FBP program (**Probe**: treatment duration, enrolment, exit criteria, the food product, patients)
- What needs improvement? Possible recommendations to improve nutritional recovery, nutritional status and overall quality of life of adult and children HIV patients.
