# Supplementary material for: Contextual and individual level factors influencing nutritional program effectiveness in HIV care setting in Tigray region, northern Ethiopia: Mixed methods study
Source: PLoS One. 2020 Apr 27;15(4):e0231859. doi: 10.1371/journal.pone.0231859 (PMC7185904; doi:10.1371/journal.pone.0231859)
Supplement: S3 File — (DOCX) [file pone.0231859.s003.docx]

| Socio demographic characteristics of study participants | | |
| --- | --- | --- |
| S.No | Questions | Code and category |
| Q101 | How old are you? | Years |
| Q102 | Gender | 1. Male 2. Female |
| Q102 | What is your religion? | 1. Orthodox 2. Muslim 3. Protestant 4. Catholic   99.Others specify |
| Q103 | What is your ethnicity? | 1. Tigray 2. Amhara 3. Oromo 4. Gurage   Others, specify |
| Q104 | What is the highest educational level attained? | 1. 1. Diploma 2. 2. BSc degree 3. 3. Masters 4. 4. Others |
| Q105 | How long has it been since you start working in your current profession | 1. ‑‑‑‑‑‑‑‑‑‑‑‑‑‑‑years |
| Q106 | What is your position in TRHB |  |
| Q107 | How long has it been since you start working in your current position? | 1. years |

1. Can you tell me a bit about the services you offered in the HIV care program?

**Probing questions**

- To the clinics in the health facilities
- To the health providers
- To the patients/clients

1. Can you describe me the specific activities of the nutrition coordinating office of the nutritional program?

- At program level
- At facility level
- Individual client

1. Ok, I am interested with the nutritional program given to HIV patients (specifically children and adults). How do you

**Probing questions**

- How do you monitor, supervise and coordinate the nutritional program

1. In your view, what health service related issues negatively or positively affect the use of the nutritional program?

**Probing questions**

- What is the level of integration of the nutritional program and other HIV services at program /regional level?
- Reporting and monitoring
- Issues related with the health facility.
- Program related issues

1. How did the nutrition coordinating office work to make nutritional counselling as a core component of the nutritional program?

**Probing questions**

- To patients
- To health facilities

1. What community related issues influence the nutritional program?

**Probing questions**

- Stigma
- Fasting

1. What do you think is the role of the nutritional program in improving retention of HIV patients in the HIV care service?( Patients adherence to the care, ART , default and retention in care)
2. As health program manager, what concerns surround the HIV care and support service in general and the nutritional program in particular? (Adult and children).

**Probing questions**

- Sustainability issue
- How continuous and linked is the nutritional program

1. Now, let’s talk about the challenges of the nutritional program, what challenges and success are there about the nutritional program? In general (adult and children in particular).

**Probing questions**

- Services or program related challenges to the FBP program (**Probe**: health system related and patient related).
- What needs improvement? Possible recommendations to improve the nutritional program and quality of life of HIV patients.
